# Integrating case-based learning into the ADDIE model for higher vocational rehabilitation nursing courses: a quasi-experimental study

**DOI:** 10.3389/fmed.2026.1864235

**Published:** 2026-08-18

**Authors:** Weihua Li, Yuhan Wang, Xiaoqiong Hu, Zhenrong Zhang, Shuangli Li

**Affiliations:** 1 School of Nursing, Chongqing Three Gorges Medical College, Chongqing, China; 2 School of Education, South-Central Minzu University, Wuhan, China; 3 Department of Orthopedics and Traumatology, Affiliated Hospital of Traditional Chinese Medicine of Chongqing Three Gorges Medical College, Chongqing, China; 4 Student Affairs Office, Chongqing Three Gorges Medical College, Chongqing, China

**Keywords:** ADDIE model, case-based learning, clinical competence, nursing, nursing education, rehabilitation nursing, students

## Abstract

**Background:**

Traditional lecture-based instruction remains predominant in higher vocational rehabilitation nursing education and emphasizes the delivery of theoretical knowledge. Passive learning and the separation of theory from practice may limit the development of students’ practical skills, critical thinking, and humanistic caring competence. Guided by the ADDIE instructional design framework, this study integrated case-based learning (CBL) to develop and evaluate an instructional model for higher vocational rehabilitation nursing education.

**Objective:**

To evaluate the effectiveness of integrating CBL with the ADDIE model (hereafter, the ADDIE-CBL integrated teaching model) in teaching rehabilitation nursing courses at vocational colleges.

**Methods:**

A quasi-experimental study design was used. Fifty-three students from the 2023 cohort of the geriatric nursing program at Chongqing Three Gorges Medical College were selected as the control group and received conventional instruction. Fifty-one students from the 2024 cohort of the same program were selected as the experimental group and received the ADDIE-CBL integrated teaching model.

**Results:**

After the intervention, theory examination scores did not differ significantly between the two groups (*p* > 0.05). The experimental group’s examination scores (regular scores, practical scores, and final scores), critical thinking disposition scores, humanistic care ability scores, and teaching satisfaction scores were higher than those of the control group (all *p* < 0.05).

**Conclusion:**

The ADDIE-CBL integrated teaching model may improve academic performance, critical thinking disposition, humanistic care ability, and student satisfaction in higher vocational rehabilitation nursing education.

## 1 Introduction

The accelerating aging of the population and the rising prevalence of chronic diseases have led to a surge in demand for rehabilitation services (1), making the training of rehabilitation nursing professionals a critical task in the healthcare sector. Research by the World Health Organization indicates that 2.4 billion people worldwide have health conditions that could benefit from rehabilitation services, yet most countries face a shortage of such services (2). In January 2026, the Chinese government proposed to “expand the supply of rehabilitation nursing and palliative care services” during the 2026–2030 period. In China, higher vocational colleges serve as the primary source of rehabilitation nursing personnel for primary-level hospitals. However, rehabilitation nursing education in these institutions remains dominated by traditional didactic teaching, creating a gap between academic training and clinical practice (3). Consequently, students lack the necessary clinical competencies in rehabilitation nursing, including practical skills, critical thinking, and patient-centered care. Therefore, reform of higher vocational rehabilitation nursing courses is imperative in the Chinese context.

In 1975, the Education Research Center at Florida State University developed the learner-centered ADDIE model, which comprises five steps: analysis, design, development, implementation, and evaluation. This structured, closed-loop instructional model has produced positive outcomes in medical education and clinical nursing training (4, 5). Case-based learning (CBL) effectively stimulates learning motivation and cultivates students’ communication and clinical decision-making skills (6), thereby facilitating the transfer of learned knowledge to clinical practice. Although the ADDIE model has been used in continuing education and undergraduate nursing programs, evidence regarding its implementation and effectiveness in higher vocational nursing education remains limited. Existing studies have largely focused on knowledge acquisition or individual skills, with less attention to broader clinical competencies such as critical thinking and humanistic caring. Moreover, previous research has generally evaluated single teaching methods rather than examining how a curriculum design framework such as ADDIE can be integrated with a specific instructional strategy such as CBL to bridge the gap between theoretical knowledge and clinical application.

Based on the above findings, we redesigned the rehabilitation nursing curriculum using the five-phase ADDIE model as the overall instructional framework. CBL was incorporated into the implementation phase as the core teaching strategy and was supported by authentic clinical cases. Layered practical activities, including role-play and standardized patient simulation, were used to create clinically relevant learning situations, and reflective assignments were incorporated to strengthen clinical reasoning and critical thinking. This multicomponent design was intended to generate complementary effects: CBL promoted active learning and knowledge application, standardized patient simulation provided contextualized skills practice, and reflective activities supported the consolidation of professional competencies.

This study therefore evaluated whether the ADDIE-CBL integrated teaching model was associated with improved academic performance, critical thinking disposition, humanistic care ability, and teaching satisfaction among higher vocational nursing students.

## 2 Materials and methods

### 2.1 Study design

**Figure 1:**
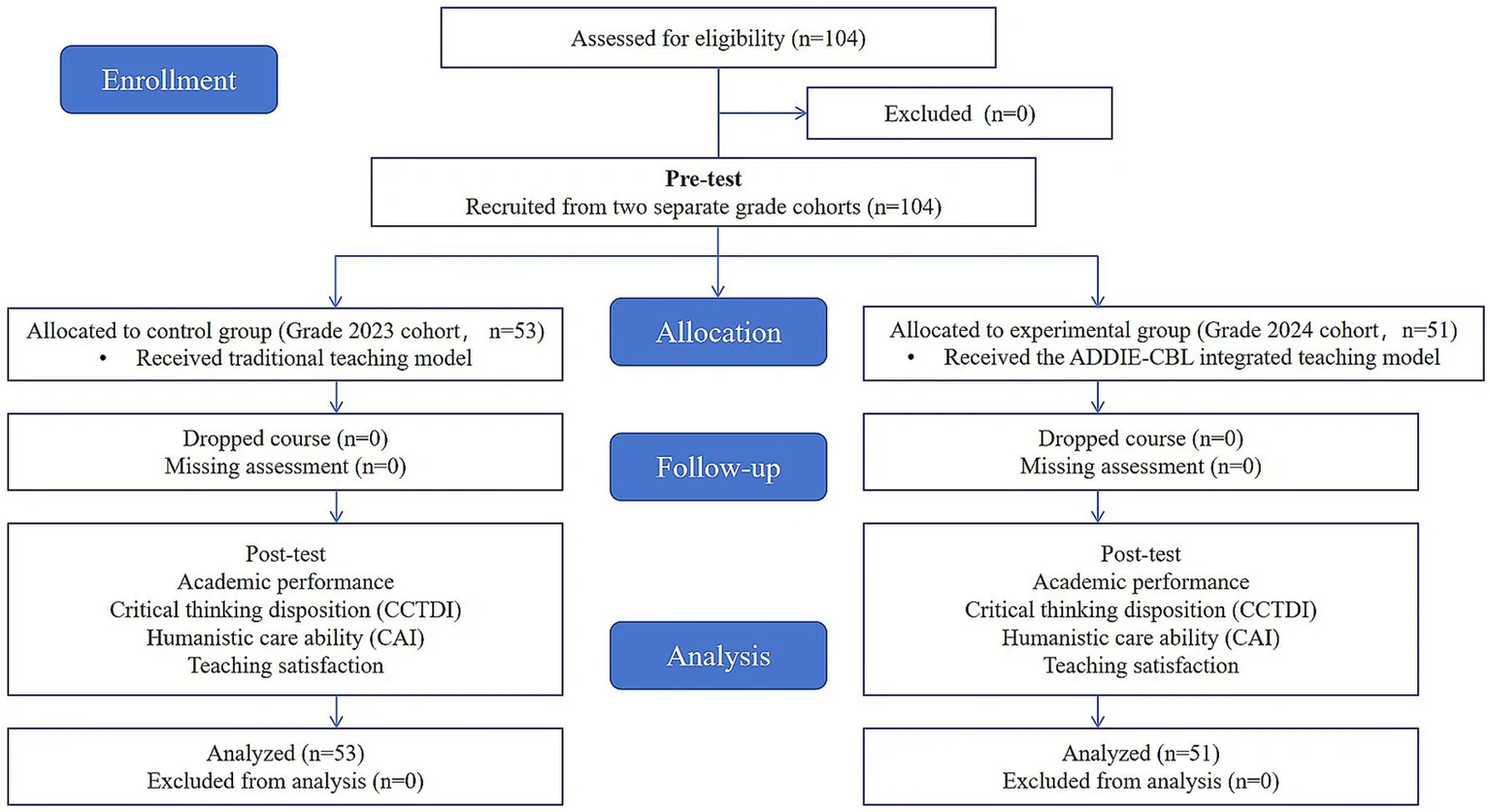
CONSORT flow diagram of the study.

This study used a quasi-experimental design. Critical thinking disposition and humanistic care ability were assessed in both groups before and after the intervention. Academic performance and teaching satisfaction were compared between the groups at the end of the course. The complete participant recruitment, grouping, follow-up and analysis process for this cross-cohort quasi-experimental study is presented in Figure 1.

### 2.2 Participants

Participants were students enrolled in the geriatric nursing program at Chongqing Three Gorges Medical College. The program was established to prepare rehabilitation nursing professionals for primary-level healthcare settings. Before the third semester of each academic year, nursing students were invited to apply voluntarily, and one class was formed according to uniform selection criteria. The study was approved by the Biomedical Ethics Committee of Chongqing Three Gorges Medical College (Ethics Approval No.: SXYZ-H-2510-0002). The inclusion criteria were as follows: (1) completion of courses in basic nursing and health assessment; (2) provision of written informed consent; and (3) voluntary participation throughout the study. The exclusion criteria were as follows: (1) withdrew from the study before completion; (2) severe physical or psychological conditions.

The study sample-size calculation for a two-tailed independent-samples *t*-test was estimated using G*Power version 3.1.9.7. It was found that when the significance level (*α*) = 0.05, power (1-*β*) = 0.80, and an effect size (*d*) = 0.60 were set (7), the minimum required sample size was 45 participants per group, for a total of 90 participants. To account for potential attrition in classroom-based studies (e.g., invalid questionnaires and absences), a 10% allowance was added, resulting in a target sample size of 99 participants, or approximately 50 participants per group. This study employed cluster sampling. Ultimately, 53 students from the 2023 cohort were included in the control group and 51 students from the 2024 cohort were included in the experimental group, exceeding the stated target sample size.

Apart from the teaching model reform evaluated in this study, the two cohorts were taught under comparable conditions. Both cohorts used the same textbook version, syllabus, assessment criteria, teaching faculty, and allocation of class time (12 theoretical hours and 20 practical hours). No major changes occurred in teaching facilities or practical training conditions. The rehabilitation nursing course was offered in the third semester for both cohorts, with the same prerequisite and subsequent courses. The institution implemented no other major pedagogical reforms or curriculum adjustments during the study period. Before the course began, the teaching team conducted joint lesson planning to standardize the course objectives and teaching tasks.

### 2.3 Research methods

#### 2.3.1 Control group

The control group followed a traditional teaching model, which involved viewing learning resources on the XuetangX platform before class and completing a pre-class assessment; during class, the instructor delivered the course content in detail and demonstrated rehabilitation nursing procedures; and after independent practice, students uploaded videos of their practical demonstrations to the XuetangX platform and completed a post-class assessment.

#### 2.3.2 Experimental group

**Figure 2:**
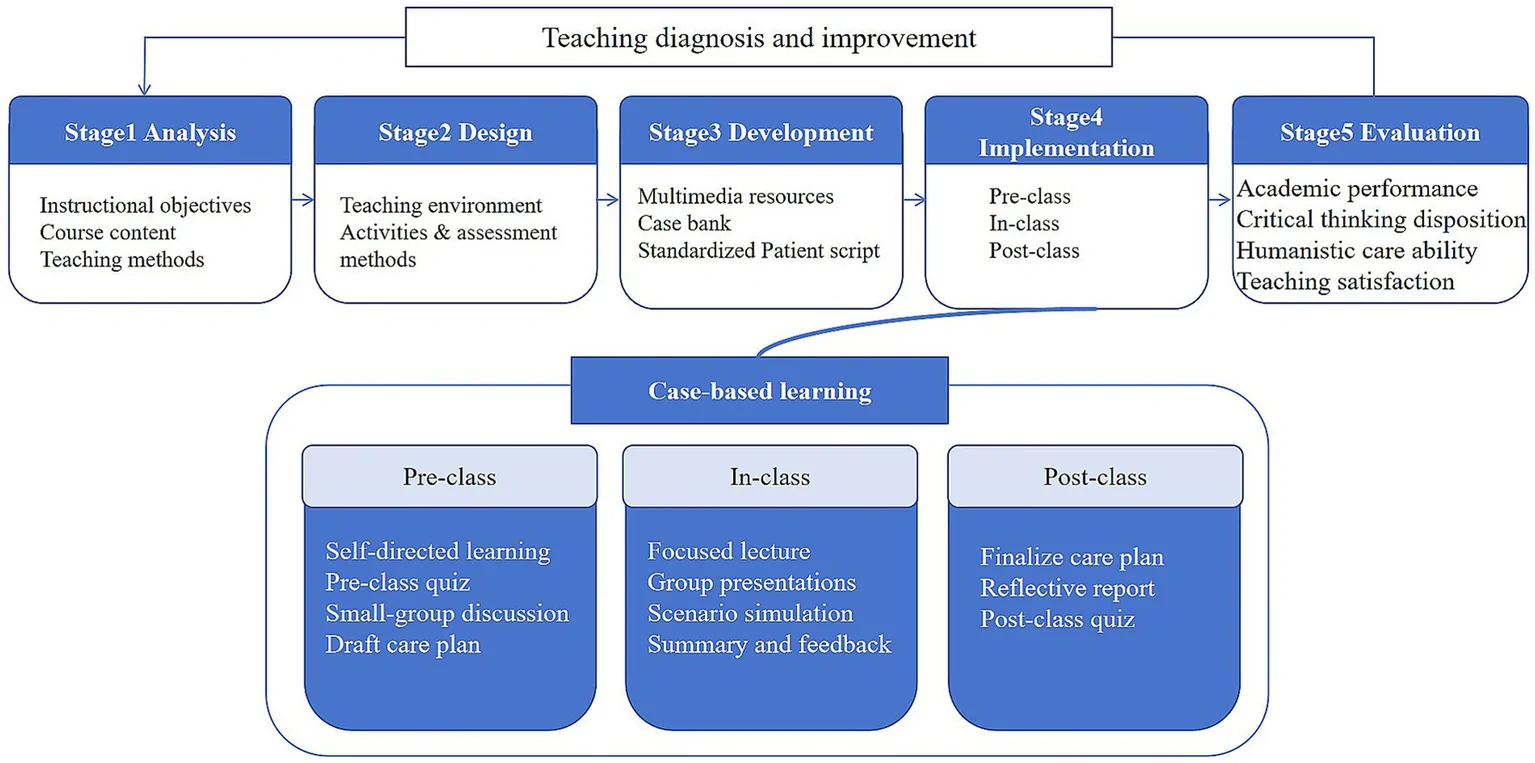
Framework of the ADDIE-CBL integrated teaching model.

The experimental group received the ADDIE-CBL integrated teaching model. The ADDIE model provided the framework for the teaching reform. The analysis and design phases defined the learning objectives, course content, and learning environment; the development and implementation phases addressed the preparation and delivery of instructional activities; and the evaluation phase assessed learning outcomes and teaching effectiveness. CBL was used as the core instructional strategy in the implementation phase to strengthen practical skills, critical thinking disposition, and humanistic care ability, and was supplemented with additional teaching approaches. The overall structure of the ADDIE-CBL integrated teaching model is illustrated in Figure 2. The details are as follows:

##### 2.3.2.1 Analysis

From June to September 2025, the research team conducted two rounds of questionnaire surveys of rehabilitation physicians, therapists, and nurses in rehabilitation departments and related clinical departments of primary hospitals across 12 provinces in China. In addition, semi-structured interviews were conducted with a selected subset of respondents. Based on the survey results, we analyzed the following three aspects: (1) Instructional objectives: What tasks are associated with rehabilitation nursing positions in primary-level hospitals? What knowledge, skills, and competencies are required of students? (2) Course content and class hours: Based on a literature review, questionnaire surveys, and semi-structured interviews, the rehabilitation nursing was designed as a 32-h course, comprising two components: (i) Basic theories of rehabilitation nursing (12 h), including an introduction to rehabilitation nursing, rehabilitation nursing assessment, common rehabilitation treatment techniques, and common rehabilitation nursing techniques; and (ii) Rehabilitation nursing for common diseases (20 h): including rehabilitation nursing for neurological disorders (8 h), musculoskeletal disorders (8 h), and chronic diseases (4 h). (3) Teaching methods: Students in the experimental group were provided with more opportunities for self-directed learning and group discussion. Given the multidisciplinary nature of the rehabilitation nursing curriculum (8), case-based and scenario-based simulation teaching methods were adopted, and students were required to complete reflective reports after class.

##### 2.3.2.2 Design

To achieve the instructional objectives and provide students with an immersive learning experience, we designed the teaching environment during this phase. (1) For theoretical teaching, we selected XuetangX as the platform for students’ self-directed online learning and used its supporting PowerPoint presentation plug-in, Rain Classroom, to facilitate blended online and face-to-face teaching. Rain Classroom supports a variety of interactive teaching activities, including random roll calls, real-time comments, and in-class quizzes. (2) For practical teaching, we transformed the rehabilitation nursing training laboratory into an environment that closely simulated a hospital rehabilitation therapy room and patient ward. The laboratory was equipped with advanced digital rehabilitation nursing equipment (including a digital occupational therapy training platform, a digital hand function training platform, and a digital upper limb function training device, among others), enabling students to learn the latest rehabilitation nursing techniques.

##### 2.3.2.3 Development

During this phase, we assembled a team of full-time faculty members and clinical rehabilitation nurses to develop systematic and diverse teaching resources for rehabilitation nursing courses. (1) Multimedia resources: We uploaded multimedia resources—including teaching slides, short videos, animations, question banks, and instructional documents—to the XuetangX platform. These resources reduced time and space constraints, enabling students to engage in self-directed learning anytime and anywhere using mobile devices, and facilitating their understanding of key knowledge points before class. (2) Case bank: Based on occupational role analysis, we identified five case themes: stroke, Alzheimer’s disease, hip replacement surgery, cervical spondylosis, and chronic obstructive pulmonary disease (COPD). We selected representative cases from the case repositories of Chongqing University Three Gorges Hospital and the Affiliated People’s Hospital of our college, and integrated them into the rehabilitation nursing teaching case bank after obtaining informed consent from the patients. Based on the teaching objectives, we transformed each medical record into a teaching case, comprising three sections: (i) Basic case information: a brief patient history, general health status, functional impairments, and psychosocial needs; (ii) Tasks: students were required to conduct a rehabilitation nursing assessment based on the case information, set rehabilitation nursing goals, develop a rehabilitation nursing plan, and evaluate the effectiveness of rehabilitation nursing; and (iii) Standardized Patient (SP) script: descriptions of the physical and psychological reactions resulting from the patient’s functional impairments and of the verbal and nonverbal communication used to express the patient’s personal, family, and social needs. By reading and analyzing the basic case information, completing the assigned tasks, meeting the SP’s needs, and evaluating the effectiveness of care, students progressively achieved the teaching objectives and developed their rehabilitation nursing competencies.

##### 2.3.2.4 Implementation

By combining the advantages of self-directed online learning on the XuetangX platform with case-based teaching, we implemented a blended learning approach consisting of three phases: (1) Pre-class: 1 week before class, students previewed relevant multimedia resources online and completed a pre-class quiz. Students were divided into study groups of 6–8 members, with one student designated as the group leader. Each group discussed the case study released in advance by the instructor and developed an initial rehabilitation care plan. One day before class, the instructor trained the SPs (i.e., students playing the roles of patients and their family members) for each group via Tencent Meeting, allowing the study groups to conduct scenario simulations in advance. If students had questions, they could consult the instructor online or refer to reference materials. (2) In-Class: First, based on the pre-class quiz results and feedback from the study groups, the instructor used mind maps to explain key points and difficult concepts thoroughly and addressed any questions. Next, the group leaders briefly presented their groups’ rehabilitation care plans for the case study; other members provided additional input, and the instructor offered a summary and feedback. After the presentations, each group conducted a scenario simulation in the rehabilitation care training laboratory. In each group, 1–2 students played the role of nurses and provided comprehensive rehabilitation care to the SPs; one student used a smartphone to take photos highlighting the group’s strengths and weaknesses and uploaded them to the Rain Classroom platform; the remaining students acted as observers and evaluated their group members, using scoring criteria that included technical skills, patient-centered care, and scenario design. The instructor circulated among the groups to provide guidance and correct errors. Finally, the observers from each study group provided feedback on their group’s performance based on the photos submitted to Rain Classroom, and the instructor offered a concluding evaluation. (3) Post-class: Each study group refined its rehabilitation care plan based on its performance in the classroom simulation and feedback from the instructor and peers, and submitted a reflective report along with the plan to the XuetangX platform. The instructor reviewed the rehabilitation care plans and reflective reports, and assessed students’ mastery of knowledge based on these materials and the post-class test results. Taking stroke rehabilitation care as an example, specific details are outlined in Tables 1, 2.

**Table 1:** The ADDIE-CBL integrated teaching model: example of stroke rehabilitation nursing.

Phase	Teaching design
Pre-class	(1) One week in advance, the instructor assigned the learning tasks to students, including previewing micro-lectures and course materials concerning “rehabilitation nursing for stroke” on the XuetangX platform and finishing the pre-class quiz. (2) Students discussed rehabilitation care issues and rehabilitation nursing measures for patients in small groups. Each student focused on one specific nursing issue and drafted a rehabilitation care plan using the following structure: nursing issue, nursing goal, nursing intervention, and nursing evaluation. (3) One day before class, the instructor conducted an online training session for students in each group who served as standardized patients and family members, covering clinical presentations, nursing needs, and verbal and nonverbal communication skills.
In-class	(1) Focused lecture: (i) The instructor used mind maps to provide a concise overview of the clinical manifestations and functional impairments associated with stroke. (ii) The instructor reviewed key points (stroke rehabilitation nursing assessment) and explained difficult concepts (key points of stroke rehabilitation nursing). (iii) The instructor answered questions that arose during the preparatory reading. (2) Group presentations: (i) The group leader presented the results of the group discussion, including the functional impairments identified from the patient’s clinical presentation; how these impairments affected the patient’s daily life and work; the assessment tools used; and the rehabilitation care plan developed from the assessment results. (ii) The instructor summarized and provided feedback on each group’s presentation, raised questions, and encouraged other group members to add details and refine their responses. (3) Scenario simulation: (i) The group leader assigned roles, and the group prepares materials and set up the scene. (ii) Students participating in the role-play advanced the scenario step by step based on the case study and the needs of the simulated patient, while student observers scored their performance. (iii) The instructor circulated among the groups to provide guidance during the simulation, corrected procedural errors, and guided students in providing comprehensive rehabilitation care to the patient. (4) Summary and feedback: (i) Students provided feedback on the images submitted via Rain Classroom. (ii) The instructor summarized each group’s performance.
Post-class	The instructor assigned the following tasks: (1) to finalize the rehabilitation care plan and upload it to the XuetangX platform; (2) to draft a group reflective report on the scenario simulation—covering the SP’s issues, corresponding interventions, strengths and weaknesses of those interventions, reasons for shortcomings, plans for future improvement, and key learning insights—and uploaded the report to the platform; and (3) to complete the post-class quiz.

**Table 2:** Design of stroke rehabilitation nursing scenarios.

Scenario	Skills and techniques	SP performance and requirements
Admission rehabilitation assessment	Motor function assessment, ADL assessment, psychological assessment	Feel down and tired
Early rehabilitation training	Proper positioning, turning exercises, joint mobility exercises, and psychological support	Express concerns about prognosis and lack confidence
Post-discharge rehabilitation and nursing education	Disease-related health education, ADL training methods, and guidance on preventing complications	Request advice on post-discharge daily life
Guidance on home rehabilitation care	Assessment of rehabilitation progress, assessment of the home environment and recommendations for modifications, guidance on the use of assistive devices	Demonstrate the use of a walking cane

##### 2.3.2.5 Evaluation

Evaluation was integrated throughout the teaching process and served to assess teaching effectiveness and promote continuous improvement in teaching quality. Observation indicators included: (1) Course examination scores: These were used to evaluate students’ mastery of rehabilitation nursing knowledge and skills, and included formative and summative assessments. Both groups used the same weighting: formative assessment accounted for 20% of the total grade, while summative assessment consisted of a practical skill examination (20%) and a theoretical examination (60%). Formative assessment results were scored automatically by XuetangX without manual scoring. Unified practical examination items and objective WorldSkills-style rubrics were used. Two trained examiners rated each student in the same laboratory, and their scores were averaged. (2) California Critical Thinking Disposition Inventory (CCTDI): The CCTDI comprises 70 items across seven dimensions—truth-seeking, open-mindedness, analytical ability, systematizing ability, confidence in critical thinking, curiosity, and cognitive maturity. It uses a 6-point Likert scale, with responses from “Strongly Disagree” to “Strongly Agree” scored from 1 to 6, respectively. The total score ranges from 70 to 420, with higher scores indicating stronger critical thinking disposition. The Chinese version of the CCTDI had a Cronbach’s alpha coefficient 0.90 and a content validity index (CVI) of 0.89 (9). (3) Caring Ability Inventory (CAI): The CAI comprises 37 items across three dimensions—understanding, patience, and courage. It employs a 7-point Likert scale, with responses ranging from 1 (“Strongly Disagree”) to 7 (“Strongly Agree”). The total score ranges from 37 to 259, with higher scores indicating stronger humanistic care ability. The Chinese version of the CAI had a Cronbach’s *α* of 0.87 and a CVI of 0.91 (10). (4) Teaching satisfaction evaluation: A teaching quality evaluation scale was self-developed by the college, comprising 12 items (example, achievement of teaching objectives, flexibility and diversity of teaching methods, appropriate use of information technology, classroom atmosphere, and improvement in students’ problem-solving and practical skills). The maximum score was 100, with higher scores indicating better teaching effectiveness. The scale has been used at the college for more than 10 years, with more than 10,000 evaluations completed annually. In the present study, the total-scale Cronbach *α* was 0.88. Ten nursing education experts rated each item on a 4-point scale; the scale-level CVI (S-CVI) was 0.92, and item-level CVIs (I-CVIs) ranged from 0.80 to 1.00. The CCTDI and CAI were administered at pretest and posttest. Baseline assessments were completed 1 week before formal teaching, and posttests were completed within 1 week after the course ended. Teaching satisfaction was also assessed within 1 week after course completion. All questionnaires were administered online, and the valid response rate was 100%.

##### 2.3.2.6 Statistical analysis

SPSS 27.0 software was used for data analysis. Independent-samples *t*-tests and Mann–Whitney *U* tests were used for continuous variables, and chi-square tests were used for categorical variables. All tests were two-tailed, with *α* = 0.05. Before conducting between-group comparisons, the Shapiro–Wilk test was used to assess the normality of continuous variables. Variables with *p* < 0.05 were considered non-normally distributed and were analyzed using the Mann–Whitney *U* test. The standardized nonparametric effect size was calculated as $r=|Z|/\sqrt{N}\;$ , where N was the total sample size. For normally distributed variables (*p* > 0.05), Levene’s test was used to assess homogeneity of variance. As the assumption of equal variances was met for all normally distributed variables (*p* > 0.05), independent-samples *t* tests were used, and Cohen’s *d* was calculated as the parametric effect size. Effect sizes were interpreted using established benchmarks (11): Cohen’s *d* values of approximately 0.20, 0.50, and 0.80 represented small, medium, and large effects, respectively; *r* values of approximately 0.10, 0.30, and 0.50 represented small, medium, and large effects, respectively. For variables analyzed using the Mann–Whitney *U* test, 95% bias-corrected and accelerated bootstrap confidence intervals for Hodges–Lehmann median differences were generated from 1,000 resamples. For normally distributed variables, 95% confidence intervals for mean differences were calculated.

## 3 Results

### 3.1 Comparison of baseline data

Comparison of baseline data between the two groups of students showed no statistically significant differences (all *p* > 0.05; Table 3).

**Table 3:** Comparison of demographic characteristics and prior-semester core-course grades between the two groups.

Group	*n*	Gender (*n*)		Average age $\left(\bar{x}\pm s\right)$	Prior education (*n*)		Mean final examination Score $\left(\bar{x}\pm s\right)$
		Male	Female		High School	Vocational High school	
Control group	53	7	46	19.401 ± 1.007	42	11	81.069 ± 8.485
Experimental group	51	9	42	19.452 ± 0.923	32	19	81.057 ± 6.609
*t/* χ ^2^		0.394		−0.289	3.447		0.008
*p*		0.530		0.773	0.063		0.994

### 3.2 Comparison of course examination scores between the two groups

As shown in Table 4, the control group’s regular grade was 90.226 ± 11.301, the theory grade was 65.642 ± 10.398, the final grade was 72.453 ± 7.683, and the median practical grade was 70.00 (P25, P75: 70.00, 80.00). The experimental group’s regular grade was 94.451 ± 2.479, the theory grade was 68.196 ± 8.893, the final grade was 76.373 ± 5.695, and the median practical grade was 83.55 (P25, P75: 81.20, 87.80). After the intervention, the experimental group’s regular, practical, and final grades were significantly higher than those of the control group (all *p* < 0.05), with medium to large effect sizes. There was no statistically significant difference in theory grades between the two groups (*p* > 0.05).

**Table 4:** Comparison of examination scores between the two groups, [ $\bar{x}\pm s$ /M(P25, P75)].

Group	*n*	Regular grade	Theory grade	Practical grade	Final grade
Control group	53	90.226 ± 11.301	65.642 ± 10.398	70.00 (70.00, 80.00)	72.453 ± 7.683
Experimental group	51	94.451 ± 2.479	68.196 ± 8.893	83.55 (81.20, 87.80)	76.373 ± 5.695
*t*/*Z*		−2.609	−1.344	−5.397	−2.947
*P*		0.010	0.182	<0.001	0.004
Cohen’s *d/r*		0.512	0.264	0.529	0.578
95% CI		−7.436, −1.013	−6.324, 1.215	−12.200, −6.000	−6.558, −1.281

### 3.3 Comparison of critical thinking scores between the two groups

As shown in Table 5, baseline comparisons showed no statistically significant differences between the two groups in any CCTDI dimension or the total score (all *p* > 0.05). At posttest, for normally distributed variables, the mean scores for the control group on truth-seeking, open-mindedness and analytical ability were 35.415 ± 8.787, 32.510 ± 4.510, 30.264 ± 6.443, respectively. The corresponding scores for the experimental group were 38.922 ± 6.980, 37.882 ± 6.562, and 32.902 ± 6.441. For non-normally distributed variables, the median scores for the control group on systematization ability, confidence in critical thinking, curiosity and cognitive maturity were 33.00 (P25, P75: 30.00, 35.00), 32.00 (P25, P75: 30.50, 37.50), 32.00 (P25, P75: 25.00, 33.50), and 34.00 (P25, P75: 30.00, 39.50), respectively. The corresponding scores for the experimental group were 37.00 (P25, P75: 32.00, 41.00), 39.00 (P25, P75: 34.00, 44.00), 37.00 (P25, P75: 35.00, 40.00), and 39.00 (P25, P75: 35.00, 43.00). For the total CCTDI score, the control group had a mean of 228.396 ± 24.388, whereas the experimental group had a mean of 259.882 ± 23.913. The scores for each dimension and the total CCTDI score were significantly higher in the experimental group than in the control group (all *p* < 0.05). Effect sizes for the seven CCTDI dimensions ranged from small to large (*d* = 0.409–0.958 for parametric variables; *r* = 0.285–0.402 for nonparametric variables), while the total CCTDI score showed a large effect (*d* = 1.303).

**Table 5:** Comparison of critical thinking scores between the two groups, [ $\bar{x}\pm s$ /M(P25, P75)].

Group	*n*	Truth-seeking		Open-mindedness		Analytical ability		Systematization ability	
		Pre-test	Post-test	Pre-test	Post-test	Pre-test	Post-test	Pre-test	Post-test
Control group	53	33.736 ± 9.011	35.415 ± 8.787	32.00 (29.50, 36.00)	32.510 ± 4.510	28.359 ± 6.174	30.264 ± 6.443	32.00 (29.50, 35.00)	33.00 (30.00, 35.00)
Experimental group	51	32.098 ± 7.455	38.922 ± 6.980	33.00 (30.00, 36.00)	37.882 ± 6.562	28.177 ± 6.128	32.902 ± 6.441	33.00 (30.00, 36.00)	37.00 (32.00, 41.00)
*t*/*Z*		1.008	−2.248	−0.623	−4.882	0.151	−2.088	−1.181	−3.538
*p*		0.316	0.027	0.533	<0.001	0.880	0.039	0.238	<0.001
Cohen’s *d*/*r*		0.198	0.441	0.061	0.958	0.030	0.409	0.116	0.351
95% CI		−1.585, 4.861	−6.601, −0.412	−2.00, 1.00	−7.556, −3.190	−2.211, 2.575	−5.144, −0.131	−3.00, 1.00	−6.00, −2.00

Group	*n*	Confidence in critical thinking		Curiosity		Cognitive maturity		Total score	
		Pre-test	Post-test	Pre-test	Post-test	Pre-test	Post-test	Pre-test	Post-test
Control group	53	31.00 (26.00, 33.50)	32.00 (30.50, 37.50)	29.00 (20.00, 33.00)	32.00 (25.00, 33.50)	32.00 (28.00, 38.50)	34.00 (30.00, 39.50)	223.00 (195.00, 237.50)	228.396 ± 24.388
Experimental group	51	31.00 (27.00, 34.00)	39.00 (34.00, 44.00)	28.00 (21.00, 33.00)	37.00 (35.00, 40.00)	32.00 (28.00, 38.00)	39.00 (35.00, 43.00)	221.00 (199.00, 233.00)	259.882 ± 23.913
*t*/*Z*		−0.010	−3.827	−0.140	−4.095	0.239	−2.903	−0.003	−6.645
*p*		0.992	<0.001	0.889	<0.001	0.811	0.004	0.997	<0.001
Cohen’s *d*/*r*		<0.001	0.375	0.014	0.402	0.047	0.285	<0.001	1.303
95% CI		−2.00, 2.00	−8.00, −3.00	−3.00, 3.00	−9.00, −3.00	−2.00, 4.00	−7.00, −1.00	−10.00, 9.00	−40.885, −22.088

### 3.4 Comparison of humanistic care ability scores between the two groups

As shown in Table 6, before the intervention, there were no statistically significant differences between the two groups in any CAI dimension or in the total score (all *p* > 0.05). At posttest, the mean scores for the control group on the dimensions of understanding, courage, and patience were 70.849 ± 11.119, 48.528 ± 10.620, and 53.623 ± 7.139, respectively, with a total score of 173.000 ± 16.123. The corresponding mean scores for the experimental group were 75.745 ± 8.800, 53.980 ± 10.000, and 56.961 ± 6.365, respectively, with a total score of 186.686 ± 21.107. After the intervention, the scores for each dimension and the total CAI score were significantly higher in the experimental group than in the control group (all *p* < 0.05), with moderate effect sizes (*d* = 0.487–0.731).

**Table 6:** Comparison of scores on humanistic care competencies between the two groups ( $\bar{x}\pm s$ ).

Group	*n*	Understanding		Courage		Patience		Total score	
		Pre-test	Post-test	Pre-test	Post-test	Pre-test	Post-test	Pre-test	Post-test
Control group	53	69.208 ± 10.104	70.849 ± 11.119	47.226 ± 8.306	48.528 ± 10.620	53.264 ± 7.440	53.623 ± 7.139	169.698 ± 16.310	173.000 ± 16.123
Experimental group	51	70.863 ± 9.527	75.745 ± 8.800	49.451 ± 8.929	53.980 ± 10.000	53.667 ± 7.369	56.961 ± 6.365	173.980 ± 19.580	186.686 ± 21.107
*t*		−0.859	−2.484	−1.316	−2.694	−0.277	−2.513	−1.214	−3.725
*p*		0.392	0.015	0.191	0.008	0.782	0.014	0.228	<0.001
Cohen’s *d*		0.168	0.487	0.174	0.528	0.054	0.493	0.238	0.731
95% CI		−5.478, 2.168	−8.806, −0.986	−5.577, 1.128	−9.466, −1.438	−3.284, 2.479	−5.973, −0.704	−11.28, 2.716	−20.975, −6.398

### 3.5 Comparison of teaching satisfaction scores between the two groups

The mean teaching quality evaluation score was 95.790 ± 4.253 in the control group and 98.468 ± 3.955 in the experimental group. The between-group difference was statistically significant (*t* = −3.305, *p* < 0.001), with a medium effect size (*d* = 0.652).

## 4 Discussion

### 4.1 The ADDIE-CBL integrated teaching model is associated with the effectiveness of rehabilitation nursing education

Table 4 shows that the experimental group’s regular grades, practical skills scores, and final grades were higher than those of the control group (all *p* < 0.05), with medium to large effect sizes. These results are consistent with those of related studies (12, 13). This study followed the five steps of the ADDIE model, and focused on analyzing the rehabilitation nursing needs of patients with common conditions. Representative clinical conditions were selected for the development of teaching cases. During implementation, the study emphasized creating a simulated clinical teaching environment and establishing progressive clinical nursing tasks from admission to discharge (rehabilitation nursing assessment → early rehabilitation nursing → discharge rehabilitation nursing education → home rehabilitation nursing). By completing tasks, solving problems, and identifying shortcomings, students’ intrinsic motivation was stimulated, encouraging active, in-depth learning and facilitating the transfer of theoretical knowledge to practical application (14). In addition, this study integrated specialized rehabilitation nursing skills (e.g., patient positioning and turning techniques) into theoretical instruction. Peer feedback and instructor guidance during practical training helped correct procedural details and reinforce weak areas (12), thereby continuously improving students’ practical proficiency. In contrast to the findings of Das et al. (15), the difference in theory scores between the two groups in this study was not statistically significant (*p* > 0.05). Two explanations are possible. First, the ADDIE-CBL model may be more strongly associated with knowledge application than with factual recall (12). Second, the course design emphasized practical skill development, consistent with the competency-based orientation of higher vocational nursing education in China.

### 4.2 The ADDIE-CBL integrated teaching model may contribute to the development of critical thinking disposition among vocational nursing students

Critical thinking is a vital skill that enables nurses to make sound clinical decisions based on patients’ conditions, reduce errors, and improve treatment outcomes (16); it is also a key objective of nursing education (17). Table 5 shows that the experimental group’s total and dimension-level critical thinking scores were significantly higher than those of the control group (all *p* < 0.05). These findings are consistent with those of other studies (18, 19), and suggest that the ADDIE-CBL integrated teaching model may contribute to the development of critical thinking disposition among higher vocational nursing students. The “One Case to the End” approach has also been reported to promote clinical reasoning and problem-solving among nursing students (20). Within a supportive learning environment, repeated case analysis may encourage students to monitor, question, and revise their reasoning. This interpretation is consistent with the literature indicating that active, case-based pedagogy can promote metacognitive awareness and self-directed learning (21). In the context of Chinese nursing education, regular clinical engagement by academic faculty may also facilitate collaboration with clinical preceptors in developing, refining, and sharing standardized case repositories across teaching settings (22).

### 4.3 The ADDIE-CBL integrated teaching model may be associated with improved humanistic care competence in vocational nursing students

Humanistic care is widely recognized as central nursing discipline and has been shown to promote patient recovery, enhance patient satisfaction, and improve overall nursing quality (23, 24). Evidence suggests that repeated humanistic care training during medical education may strengthen humanistic competence in subsequent clinical practice (25). Experiential approaches may be particularly relevant when students encounter patient distress, suffering, or end-of-life concerns (26). In the present study, the experimental group had higher posttest total and dimension scores on the CAI than the control group (Table 6). These findings suggest that the ADDIE-CBL model may support caring ability among higher vocational nursing students. Guo (27) proposed a four-stage model of humanistic nursing education comprising scenario setting, emotional triggering, practical experience, and reflective instruction. Consistent with this framework, the present intervention included case-based discussion, role-play, assuming the patient role during simulation, and structured sharing of experiences and feedback. These activities may have progressively deepened students’ understanding of humanistic caring and supported its translation into clinical behavior.

### 4.4 The ADDIE-CBL integrated teaching model may contribute to improve teaching satisfaction

The teaching quality evaluation score was higher in the experimental group than in the control group (*p* < 0.05). Traditional hands-on training follows a teacher-led three-step process: the teacher demonstrates, the students practice, and the teacher evaluates. In the present study, multiple rehabilitation nursing procedures were integrated into a comprehensive case scenario, thereby linking practical skills with the clinical context. This design gave students additional opportunities for repeated practice and reflection, which may have contributed to stronger perceived clinical competence and higher teaching satisfaction (28). Nevertheless, mean satisfaction scores exceeded 95 in both groups, suggesting a possible ceiling effect. This finding may reflect a general tendency toward highly favorable ratings on teaching evaluations in this setting. Future studies should use mixed-methods evaluations that combine quantitative and qualitative indicators to provide a more multidimensional assessment of teaching interventions.

## 5 Limitations and recommendations

This study has several limitations. First, the nonrandomized comparison of two consecutive cohorts may have introduced cohort effects and selection bias. A stronger design would recruit parallel classes from the same cohort and use random allocation when feasible. Second, the absence of a between-group difference in theory examination scores suggests that the ADDIE-CBL model may be better suited to practice-oriented higher vocational nursing courses, such as Fundamentals of Nursing and Gerontological Nursing. Further research should examine its applicability across a broader range of nursing courses. Third, outcomes were assessed immediately after course completion; whether improvements in critical thinking disposition, caring ability, and practical skills are sustained remains unknown. Follow-up assessments at 6 or 12 months are needed to evaluate longer-term effects. Finally, although in-class teaching time was equivalent across cohorts, the multicomponent intervention increased the experimental group’s out-of-class learning workload, creating a potential confounder. Future controlled studies should equalize total learning time and workload across groups.

## 6 Conclusion

The ADDIE model provides a clear framework and pathway for educators in higher vocational nursing education to implement instructional reforms. Under the conditions of the present study, the ADDIE-CBL integrated teaching model was associated with improvements in students’ practical skills, critical thinking disposition, and humanistic care ability, but not in theoretical knowledge acquisition. This pattern suggests that the model’s primary strength lies in facilitating knowledge application and higher-order thinking, rather than enhancing factual recall. As such, the ADDIE-CBL model may serve as a promising alternative to conventional teaching methods for practice-oriented nursing courses.

## Data Availability

The raw data supporting the conclusions of this article will be made available by the authors, without undue reservation.
